# Muscle Synergies in Cycling after Incomplete Spinal Cord Injury: Correlation with Clinical Measures of Motor Function and Spasticity

**DOI:** 10.3389/fnhum.2015.00706

**Published:** 2016-01-11

**Authors:** Filipe O. Barroso, Diego Torricelli, Elisabeth Bravo-Esteban, Julian Taylor, Julio Gómez-Soriano, Cristina Santos, Juan C. Moreno, José L. Pons

**Affiliations:** ^1^Electronics Department, University of MinhoGuimarães, Portugal; ^2^Neural Rehabilitation Group, Cajal Institute, Spanish National Research CouncilMadrid, Spain; ^3^Sensorimotor Function Group - Hospital Nacional de Parapléjicos, Servicio de Salud de Castilla la Mancha (SESCAM)Toledo, Spain; ^4^iPhysio Research Group, San Jorge UniversityZaragoza, Spain; ^5^National Spinal Injuries Centre, Stoke Mandeville Spinal Research, Buckinghamshire Health Trust, National Health Service (NHS)Aylesbury, UK; ^6^Harris Manchester College, University of OxfordOxford, UK; ^7^Toledo Physiotherapy Research Group (GIFTO), Nursing and Physical Therapy School, Castilla la Mancha UniversityToledo, Spain

**Keywords:** muscle synergies, spinal cord injury, cycling, spasticity, motor function

## Abstract

**Background:** After incomplete spinal cord injury (iSCI), patients suffer important sensorimotor impairments, such as abnormal locomotion patterns and spasticity. Complementary to current clinical diagnostic procedures, the analysis of muscle synergies has emerged as a promising tool to study muscle coordination, which plays a major role in the control of multi-limb functional movements.

**Objective:** Based on recent findings suggesting that walking and cycling share similar synergistic control, the analysis of muscle synergies during cycling might be explored as an early descriptor of gait-related impaired control. This idea was split into the following two hypotheses: (a) iSCI patients present a synergistic control of muscles during cycling; (b) muscle synergies outcomes extracted during cycling correlate with clinical measurements of gait performance and/or spasticity.

**Methods:** Electromyographic (EMG) activity of 13 unilateral lower limb muscles was recorded in a group of 10 healthy individuals and 10 iSCI subjects during cycling at four different cadences. A non-negative matrix factorization (NNMF) algorithm was applied to identify synergistic components (i.e., activation coefficients and muscle synergy vectors). Reconstruction goodness scores (VAF and *r*^2^) were used to evaluate the ability of a given number of synergies to reconstruct the EMG signals.

A set of metrics based on the similarity between pathologic and healthy synergies were correlated with clinical scales of gait performance and spasticity.

**Results:** iSCI patients preserved a synergistic control of muscles during cycling. The similarity with the healthy reference was consistent with the degree of the impairment, i.e., less impaired patients showed higher similarities with the healthy reference. There was a strong correlation between reconstruction goodness scores at 42 rpm and motor performance scales (TUG, 10-m test and WISCI II). On the other hand, the similarity between the healthy and affected synergies presented correlation with some spasticity symptoms measured by Penn, Modified Ashworth and SCATS scales.

**Conclusion:** Overall, the results of this study support the hypothesis that the analysis of muscle synergies during cycling can provide detailed quantitative assessment of functional motor impairments and symptoms of spasticity caused by abnormal spatiotemporal muscle co-activation following iSCI.

## Introduction

The recovery of walking functions is one of the main goals of rehabilitation of subjects with SCI (van Middendorp et al., 2014). In this regard, one of the most promising directions toward tailored interventions is the definition of reliable metrics for the assessment of functional recovery on a quantitative and fine-grained level. The main drawback of the current clinical scales is the inability to grasp modest changes in neurophysiological or functional improvements (Loftus, 2008; Kirshblum et al., 2011; Gómez-Soriano et al., 2012; Bravo-Esteban et al., 2014), due to the difficulty of measuring the complex interplay of the impaired mechanisms emerging after the lesion (Gómez-Soriano et al., 2010; Kennedy et al., 2012; Bravo-Esteban et al., 2013; van Middendorp et al., 2014). Among these, the development of the spasticity syndrome is one of the crucial contributors for motor impairment (Bravo-Esteban et al., 2013). Although the most common clinical definition of spasticity is based on the velocity-dependent properties of stretch reflex activity (Lance, 1980), this syndrome involve other symptoms such as hypertonia, spasms, clonus, hyperreflexia and muscle co-activation, all these correlated with dysfunctional muscle activity (Burridge et al., 2005; Dietz and Sinkjaer, 2007; Bennett, 2008; Arene and Hidler, 2009; Rosa et al., 2014). As such, an improvement on the estimation of muscle activity may represent an important outcome measure of motor recovery after SCI (Awai and Curt, 2014). In particular, the analysis of multi-joint muscle activation would support clinical decisions based on residual useful motor function, help to assess the effects of standard and novel therapies, and guide the prescription of standard anti-spastic medications (Bowden and Stokic, 2009; Reichenfelser et al., 2012).

The analysis of muscle synergies has been referred to as a reliable method to estimate muscle coordination and to gain insights into motor control mechanisms in both healthy and neurologically affected individuals (d'Avella and Bizzi, 2005; Bizzi et al., 2008; Clark et al., 2010; Cheung et al., 2012; Berger et al., 2013; Oliveira et al., 2014). The concept of muscle synergy has been used with different meanings, depending on the context. In neurorehabilitation, it refers to the stereotyped muscle activation observed after lesions and due to a loss of independent control, which results in abnormal and less flexible movement repertoires (Howle, 2002). In contrast, in motor neuroscience it indicates a strategy to simplify the motor control, in which a set of muscles are organized in functional groups (Clark et al., 2010). In the last 15 years, the neuroscientific perspective has gained more and more relevance and will also be adopted along this study. More specifically, this study will adopt the hypothesis of “synchronous synergies” (Tresch and Jarc, 2009), in which groups of muscles are activated in a fixed balance to produce motor tasks. Within this hypothesis, every time a synergy is activated, all the muscles within that same synergy will be active.

Recent studies in healthy subjects demonstrated that the combination of three to four muscle synergies can explain most of the activation of lower limb muscles during walking and cycling (Clark et al., 2010; Hug et al., 2010, 2011; Gizzi et al., 2011; De Marchis et al., 2012; Barroso et al., 2013, 2014). In the case of poststroke patients, the number of muscle synergies have been related to gait performance and proposed as a potential alternative to gold-standard clinical scales (Clark et al., 2010; Routson et al., 2013). In SCI patients, it has been also observed that a disruption of muscle coordination plays a major role in functional performance of overground walking (Hayes et al., 2014). In particular, Ivanenko et al. (2003) showed that muscle synergies of less impaired SCI subjects were more similar to the healthy reference than those of the more affected patients.

During the early stage of rehabilitation after the lesion, the measurement of the gait-related motor abilities is particularly difficult to be performed, due to the lack of the required muscle strength to perform rhythmic movements while maintaining appropriate upright posture. To solve this problem, we hypothesized that cycling might be explored as a novel tool for the measurement of gait-related motor performance, based on the similar synergistic control of walking and cycling in healthy controls (Barroso et al., 2014).

This paper aims to test more directly whether pedaling may be used to assess gait performance in patients with iSCI. To this regard, we formulated two further hypotheses: (i) synergistic control of muscles is preserved during cycling after iSCI. (ii) muscle synergies extracted during cycling correlate with clinical measurements of gait performance and/or spasticity. The first hypothesis, apart from being a prerequisite for testing the second hypothesis, has not been investigated previously in the literature, being most of the previous studies on muscle synergies focused on locomotion. The second hypothesis incorporates a clinically oriented question: which is the most efficient experimental setup to extract reliable results in clinical context? To answer this question, we included in our experimental design different cycling velocities and number of muscles, representing the variables that mostly affect the duration of a typical clinical assessment procedure.

## Materials and methods

### Subjects

All recruited subjects gave their written consent to participate in the study and for data publication, after being informed about the procedures and possible discomfort associated with the experiments, in accordance with the Declaration of Helsinki. The local Toledo Paraplegics Hospital Clinical Ethical Committee approved this study (07/05/2013 N°47). Ten healthy subjects (6 men and 4 women), with an age of 33.9 ± 8.48 year (25.75 – 44.5, 25th percentile—75th percentile), with no diagnosed neural injury, neither central nor peripheral, were recruited to participate as controls. Ten iSCI patients (6 men and 4 women), with an age of 43.08 ± 14.32 year (25.69 – 59.31), 7.23 ± 4.86 months (2.72 – 9.66) post-SCI, volunteered to participate in this study. All of them received the standard rehabilitation program of the hospital. Inclusion criteria were: aged between 18 and 80 year; motor incomplete spinal lesion (AIS C-D) of traumatic and non-traumatic etiology, with prognosis of recovery of the walking function; evolution of at least 1.5 months. Exclusion criteria were: supraspinal or peripheral neurological involvement; history of epilepsy; musculoskeletal involvement of lower limbs or spasticity higher than 3 (measured with the Modified Ashworth Scale) for each joint, either for extension or flexion. Detailed information of the patients is presented in Table 1.

**Table 1:** **Individual iSCI patients' description**.

**Patient ID**	**Age (Years)**	**Gender**	**Time post-SCI (months)**	**Level of Lesion**	**Most affected side**	**AIS**
1	25	M	9	C5	Right	C
2	46	M	6	T9	Left	D
3	61	M	4	T10	Right	D
4	25	M	5	T4	Left	D
5	37	M	25	C3	Left	D
6	19	F	2	C6	Right	D
7	36	F	3	T3	Left	D
8	58	F	2	C5	Right	D
9	77	M	13	C7	Right	D
10	44	F	5	C4	Left	C

*M, male; F, female; Level of Lesion: C, Cervical, T, Thoracic; AIS, American Spinal Injury Association (ASIA) Impairment Scale*.

### Experimental protocol

Prior to the experiment, a trained physiotherapist performed a set of clinical evaluations in order to inform about the clinical and functional status of the patients. The hypertonia of the muscles of the ankle and knee joints was evaluated using the modified Ashworth scale (MAS, see Table 2; Bohannon and Smith, 1987). The frequency of spasms was assessed using the Penn scale (Penn et al., 1989). Patients were also assessed with the Spinal Cord Assessment Tool for Spastic Reflexes (SCATS), which measures three types of spastic reflexes in SCI patients: clonus, flexor spasms and extensor spasms, each of them rated from 0 (no reaction) to 3 (severe; Benz et al., 2005). Patients showing a Total MAS higher than 1 or a Penn score greater or equal to 1 were characterized as presenting the spasticity (see Table 2), as done by Bravo-Esteban et al. (2014).

**Table 2:** **Amount of physical assistance needed, gait performance, and spasticity syndrome scores of iSCI patients**.

**Patient ID**	**WISCI II**	**TUG (s)**	**10-m (s)**	**MAS**				**MAS Knee**	**MAS Ankle**	**Total MAS**	**Penn scale**	**SCATS**		
				**KF**	**KE**	**DF**	**PF**					**C**	**F**	**E**
1^†^	16	22.0	23.0	1	1	1	1	2	2	4	2	1	0	1
2	20	23.0	12.0	0	0	0	0	0	0	0	0	0	0	0
3^†^	16	44.7	50.8	0	1+	1+	0	2	2	4	1	1	0	0
4^†^	15	29.3	27.7	2	1	3	0	4	4	8	1	1	0	1
5^†^	20	N.A.	N.A.	0	1+	3	0	2	4	6	1	0	1	1
6^†^	0	N.A.	N.A.	1+	2	3	0	5	4	9	2	3	1	0
7^†^	16	31.0	30.0	1+	0	1+	0	2	2	4	0	2	0	0
8	13	27.0	23.0	0	0	0	0	0	0	0	0	0	0	0
9	19	25.1	10.2	0	0	0	0	0	0	0	0	0	0	0
10^†^	8	N.A.	N.A.	0	0	3	0	0	4	4	3	3	0	3

WISCI II, Walking Index for Spinal Cord Injury; TUG, Timed Up and Go; MAS, Modified Ashworth Scale; KF, knee flexion; KE, knee extension; DF, dorsiflexion; PF, plantarflexion. MAS Knee, sum of KF and KE. In order to sum, 1+ counts as 2, 2 counts as 3, 3 counts as 4 and 4 counts as 5; MAS Ankle, sum of DF and PF; Total MAS, sum of MAS Knee and MAS Ankle; Penn, Penn scale; SCATS, Spinal Cord Assessment Tool for Spastic Reflexes; Types of spastic reflexes: C, clonus; F, flexion; E, extension; N.A., measure not available;

† , *patients characterized as presenting the spasticity syndrome*.

The gait performance of seven out of the ten iSCI patients was evaluated using the Timed Up and Go (TUG) test (Wall et al., 2000) and the 10-m test (Forrest et al., 2014). The other three patients were unavailable to perform these tests. The Walking Index for Spinal Cord Injury (WISCI II) was used to assess the amount of physical assistance needed by the patients to walk 10 m. This is a 21-point scale that ranges from 0 (patient unable to stand and/or participate in assisted walking) to 20 (patient ambulates 10 m with no devices, no braces and no physical assistance; Dittuno and Ditunno, 2001).

On the day of the experiment, patients received their standard rehabilitation therapy in the morning, and performed the cycling trials in the afternoon. For both iSCI patients and healthy subjects, four cycling trials (at 30, 42, 50, and 60 rpm, revolutions per minute) of 30 s duration each, with 60 s resting between trials, were performed on an electronically braked cycle ergometer (MOTOmed viva2, Reck, Betzenweiler, Germany). For each participant, the order of the trials was randomized to avoid biased results. All participants were asked to perform the experiment while sat on a regular chair. Patients who felt more comfortable doing the experiments on their own wheelchair were allowed to remain sat on it. When this occurred, a pillow was placed on the backside to maintain the pedaling position similar to the regular chair position.

An auditory metronome was used to synchronize participants' cycling frequency with the desired cadence. The cycling resistance (gear) of the ergometer was individually chosen by each iSCI patient, in order to cycle comfortably with the same gear across the four cadences. A constant gear was set for all control subjects.

### Data collection

A surface electromyography (sEMG) acquisition system (EMG-USB, OT Bioelettronica, Torino, Italy) was used to record the activity of 13 muscles of the most affected leg of patients, and the dominant leg of healthy subjects. The recorded muscles were: Gluteus Medius (GMe), Adductor Longus (AL), Sartorius (Sar), Tibialis Anterior (TA), Rectus Femoris (RF), Tensor Fascia Latae (TFL), Vastus Lateralis (VL), Vastus Medialis (VM), Biceps Femoris (BF), Semitendinosus (Sem), Soleus (Sol), Gastrocnemius Lateralis (GaL), and Gastrocnemius Medialis (GaM). We determined the most affected side of each patient based on the muscle score (Seddon, 1976) of quadriceps, hamstrings, TA and gastrocnemius for both limbs. The EMG amplifier had a recording bandwidth of 10–750 Hz, overall gain of 1000 V/V, and acquisition frequency of 2048 Hz. SENIAM recommendations for sEMG recording procedures (Hermens et al., 1999) were followed before placing the electrodes: shaving the places where the electrodes were placed; cleaning the skin with alcohol to minimize impedance; allowing the alcohol to vaporize in order to dry the skin before placing the electrodes. After that, bipolar electrodes (Ag-AgCl, AmbuNeuroline 720, Ambu, Ballerup, Denmark) were fastened with a 2-cm interelectrode distance on each recorded muscle. Finally, the electrodes and cables were wrapped with bandages to ensure that the wires did not impede cycling movements and also to avoid movement-induced artifacts. Preliminary tests were performed to check for cross-talk and contact artifacts, giving special attention to the hamstrings of those patients that cycled on their own wheelchair. When needed, electrodes were repositioned.

Crank angle was measured with a potentiometer (Vishay, Malvern, PA), and digitalized with a sampling frequency of 100 Hz. The bottom dead center (BDC) position of the pedal of the analyzed leg was used to segment data into pedaling cycles. EMG and angular data were synchronized by means of a common synchronization signal.

Data were analyzed offline with MATLAB R2011a (The MathWorks, Natick, MA) and IBM SPSS Statistics 20 software (IBM).

### Muscle synergies extraction

For each trial and participant, the ten consecutive central cycles were selected for analysis. The selected raw EMG signals were high-pass filtered at 20 Hz, demeaned, rectified, and smoothed with a low-pass filter at 5 Hz, resulting in the EMG envelopes (Clark et al., 2010; Hug et al., 2010; Barroso et al., 2014). EMG envelopes from each muscle were then normalized by the average of the maximum of each of the ten cycles, and resampled at each 1% of the cycling cycle. For each participant and trial, normalized EMG envelopes were combined into an *m* × *t* matrix (EMG_0_), where *m* is the number of muscles (thirteen in this case) and *t* is the time base [*t* = no. of cycles (10) × 100)].

Differences between mean EMG envelopes of iSCI patients and the mean EMG envelopes of the healthy group were assessed with the r_max_ coefficient (Hug et al., 2011), which is the maximum of the cross-correlation between two signals. The cross-correlation is calculated by the MATLAB xcorr function for centered data (option = “coeff”) and the output values as the maximum of the cross-correlation function, which gives an indication of the similarity of shape of the EMG envelopes.

Muscle synergies were extracted using the Non-Negative Matrix Factorization (NNMF) algorithm (Lee and Seung, 1999). Mathematically, the output of the algorithm is the following:

(1) $$\begin{array}{r} \text{EMG}0=\text{WH}+e=\text{EMGr}+e \end{array}$$

where W is a *m* × *n* matrix (*n* is the number of synergies) that specifies the relative weighting of each muscle within each synergy (hereafter, each column of W will be referred to as *muscle synergy vector*); H is a *n* × *t* matrix that specifies the time-varying *activation coefficients*, which represent the recruitment of each synergy vector over time; EMG_r_, is an *m* × *t* matrix resulting from the multiplication of W and H, representing the reconstructed EMG envelopes; *e* is the residual error. For each EMG_0_, we run the NNMF algorithm four times, considering as input 2 to 5 synergies (*n* = 2, 3, 4, 5). In order to avoid local minima, for each run, the NNMF was repeated 40 times and the repetition with the lowest reconstruction error was selected.

The similarity between EMG_0_ and EMG_r_ was calculated based on two reconstruction goodness scores: the *variability accounted for* (VAF_total_; Clark et al., 2010; Barroso et al., 2014) and the *coefficient of determination* (*r*^2^; Torres-Oviedo et al., 2006). VAF_total_ is described in Equation (2).

(2) $${\text{VAF}}_{\text{total}}=1-\;\frac{\sum_{i=1}^{m}\sum_{j=1}^{t}{\left(EMG_{0}\left(i,j\right)-EMG_{r}\left(i,j\right)\right)}^{2}}{\sum_{i=1}^{m}\sum_{j=1}^{t}{\left(EMG_{0}\left(i,j\right)\right)}^{2}}$$

The coefficient of determination was calculated by the MATLAB function “rsquare.” VAF was also computed for each muscle individually (VAF_muscle_). Both VAF_total_ and *r*^2^ have been adopted in most studies on muscle synergies (Torres-Oviedo et al., 2006; Clark et al., 2010; Barroso et al., 2014). VAF_total_ has been suggested to be more stringent than *r*^2^, since it is sensitive to both shape and amplitude of the signals, whereas *r*^2^ only addresses similarity in shape.

Each muscle synergy vector (column of matrix W) was normalized by the maximum value of the muscle in the synergy to which they belong, and the corresponding activation coefficients (lines of matrix H) were scaled by the same quantity (De Marchis et al., 2012; Barroso et al., 2014).

For each cadence, we obtained a reference set of matrices (hereafter called W_0_ and H_0_) by pooling the EMG envelopes from all the healthy subjects, and applying the NNMF algorithm, constrained to a fixed number of synergies. This fixed number was defined as the minimum number of synergies needed to obtain VAF_total_ values ≥ 85% in at least half of the healthy participants. Synergy vectors of each patient were ordered according to their similarity with columns of W_0_, at the corresponding matched speeds. This was done by means of the normalized scalar product (Barroso et al., 2014). After being ordered, muscle synergy vectors and activation coefficients of each patient were compared with the reference healthy set (W_0_ and H_0_) using the normalized scalar product.

**Figure 1:**
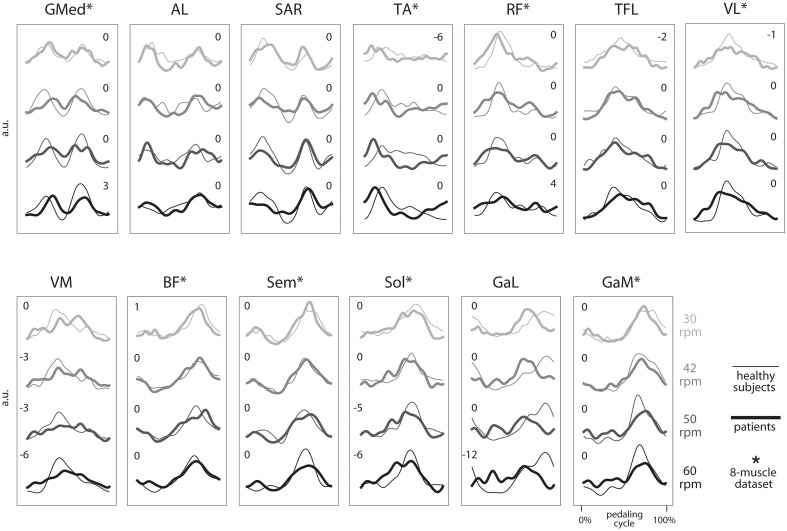
**Group average electromyographic (EMG) envelopes of the 13 recorded muscles for each of the four speeds during cycling (30, 42, 50, and 60 rpm)**. For each group (healthy subjects, thin lines; iSCI patients, thick lines), a total of 100 cycling cycles (10 cycles by subject) were averaged and expressed as a function of the pedaling cycle. Pedaling cycle begins when the pedal corresponding to the dominant leg (in healthy subjects) or the most affected leg (in iSCI subjects) is at the lowest position and ends when the pedal reaches the lowest position again. EMGs from each subject and muscle were previously normalized by the average of its maximum values throughout the 10 cycles. a.u., arbitrary unit. ^*^, muscles belonging to the eight muscles set used in parallel analysis. Number indicate lag times that maximized the cross-correlation function. A negative value indicates that the mean EMG envelopes of iSCI patients shifted earlier in the cycle relative to the mean EMG envelopes of healthy group.

In order to study the sensitivity of the method to the number of muscles, we repeated the overall process on two muscle datasets, the first including all (13) muscles recorded in the experiment, and the second including the eight muscles considered in our previous study (Barroso et al., 2014). These eight muscles are marked with an asterisk in Figure 1.

### Statistical analysis

Independent Student's *t*-tests were computed to test the similarity between the cadences achieved by both groups, at each matched speed. Independent Student's *t*-tests were also performed to test the similarity of the VAF_total_ and *r*^2^ scores between the two groups and between spastic and non-spastic patients, when using 2 to 5 synergies.

Spearman's Rank-Order correlations (*r*_s_) were performed to evaluate the monotonic relationship between reconstruction goodness scores (obtained for each different number of muscle dataset and speed) and gait performance scales (WISCI II, TUG and 10-m tests). Spearman's Rank-Order correlations were also used to evaluate the relationship between the normalized scalar products between H and H_0_ and between W and W_0_ (hereafter denoted with “H · H_0_” and “W · W_0_”) and spasticity scales scores (Total MAS, Penn and spastic reflexes from SCATS).

Statistical significance was set by a *p*-value of 0.05.

## Results

### Muscle synergy analysis

#### Cadence

SCI group cycled with a cadence of (mean ± SD) 30.26 ± 0.87, 41.89 ± 1.04, 49.71 ± 1.91, and 57.73 ± 3.57 rpm for the desired speeds of 30, 42, 50, and 60 rpm, respectively. The healthy group cycled with a cadence of 30.21 ± 0.34, 42.21 ± 1.15, 49.25 ± 0.76, and 59.79 ± 0.90 rpm, respectively. The tests of equality of means from Independent Student's *t*-tests revealed no significant differences between the two groups, in any of the four speeds considered. In particular, *p*-values of 0.868, 0.516, 0.492, and 0.093 were obtained for speeds of 30, 42, 50, and 60 rpm, respectively.

#### Individual EMG profiles

The average EMG envelopes of each group, for each of the 13 recorded muscles, are represented in Figure 1. Average activations of GMed, AL, Sar, TA and, to a lower extent, RF, occurred during the initial upstroke phase. Average activations of TFL, VL, and VM were observed during the final upstroke phase and initial downstroke. Average activation of BF, Sem, Sol, GaL, and GaM occurred mostly during the downstroke phase of cycling.

For each muscle, the shape and activation timing of average EMGs were very similar across speeds and also between the two groups, except for TA, VM, Sol, and GaL. In the case of these four muscles, the timing of maximum activation occurred earlier in the pedaling cycle in the iSCI patients, as indicated by the lag times in Figure 1. On the other hand, maximum values of the cross-correlation function were lower for Sol and GaL, when compared with the other muscles. For these two muscles, maximum correlations were lower than 0.85, while maximum correlations were higher than 0.95 for the other muscles at most of the speed conditions.

#### Reconstruction goodness scores

According to our criterion defined previously (VAF_total_ values ≥ 85% for at least half of the healthy participants), three synergies were sufficient to describe most of the variance of the two different sets of muscles in the two groups of participants analyzed. For this reason, our analysis along the paper is based on this number of synergies.

**Figure 2:**
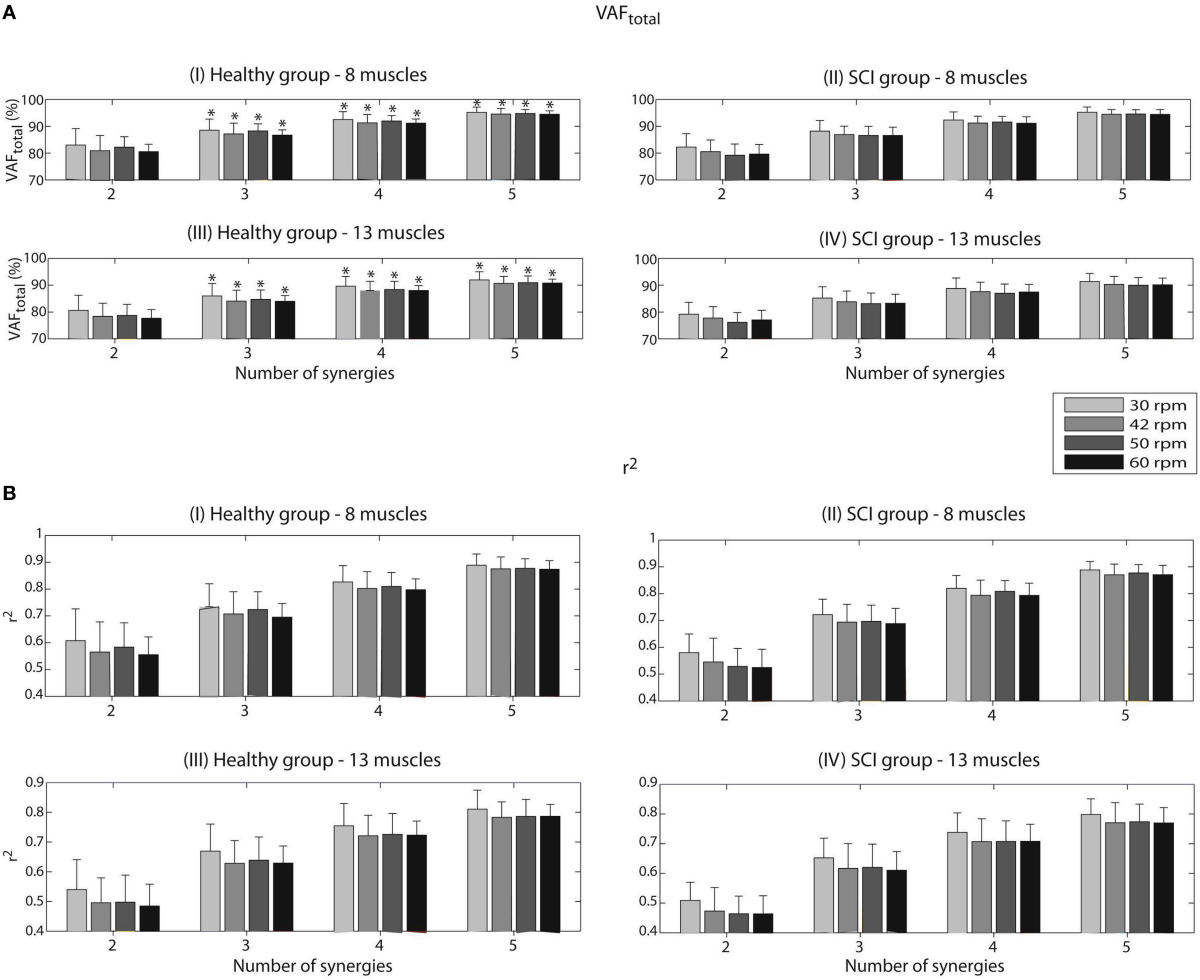
**Variability accounted for (VAF_total_) (A) and coefficient of determination (*r*^2^) (B) according to the number of synergies, for each of the four speeds (30, 42, 50, and 60 rpm)**. Values are given in means ± SD. These reconstruction goodness indexes were calculated after running the NNMF algorithm to reconstruct a set of 8 EMG envelopes for the healthy group (I) and the iSCI group (II), as well as a set of 13 EMG envelopes (III and IV for the healthy group and iSCI group, respectively). A VAF_total_ value of 100% and a *r*^2^ value of 1 mean perfect reconstruction of the EMG set. ^*^Number of synergies sufficient to describe VAF_total_ values ≥ 85% for at least half of the healthy participants.

For the set of 8 muscles, both groups reached their minimum values of VAF_total_ at the speed of 60 rpm. The VAF_total_ values in such condition were 86.7 ± 2.0% (Figure 2AI) for the healthy group and 86.6 ± 3.1% (Figure 2AII) for the iSCI group. The maximum VAF_total_ was obtained at the speed of 30 rpm, reaching values of 88.5 ± 4.2% and 88.2 ± 3.9% for the healthy and iSCI groups, respectively.

When analyzing VAF_muscle_ values with three synergies for the set of 8 muscles, all the muscles presented values higher that 75%. For instance, in the case of the healthy group, a minimum VAF_muscle_ value of 83.0 ± 13.0% was obtained for Sem at the speed of 30 rpm; a maximum VAF_muscle_ value of 91.9 ± 4.7% was obtained for VL at the speed of 50 rpm. In the case of the iSCI group, a minimum VAF_muscle_ value of 75.6 ± 8.4% was obtained for GMe at the speed of 60 rpm; a maximum VAF_muscle_ value of 91.0 ± 2.4% was obtained for GaM at the speed of 30 rpm.

VAF_total_ values decreased when considering the complete set of 13 muscles. The minimum values of 84.0 ± 2.2% (Figure 2AIII) and 83.1 ± 4.0% (Figure 2AIV) were obtained for the healthy and iSCI group, respectively. In this case, the healthy group reached minimum VAF_total_ values at the speed of 60 rpm, whereas iSCI group reached its minimum at the speed of 50 rpm. The maximum values of 86.0 ± 4.6% and 85.2 ± 4.2% were obtained for the healthy and iSCI group, respectively, both at 30 rpm.

When analyzing VAF_muscle_ values with three synergies for the set of 13 muscles, all the muscles presented values higher that 75%, except for one muscle and condition in the iSCI group. Specifically for the case of iSCI group, a minimum VAF_muscle_ value of 73.8 ± 10.8% was obtained for GMe at the speed of 60 rpm; a maximum VAF_muscle_ value of 87.4 ± 3.3% was obtained for BF at the speed of 60 rpm. In the case of the healthy group, a minimum VAF_muscle_ value of 77.2 ± 11.1% was obtained for AL at the speed of 42 rpm; a maximum VAF_muscle_ value of 91.9 ± 4.6% was obtained for VL at the speed of 30 rpm.

The tests of equality of means from Independent Student's *t*-tests revealed no significant differences for the VAF_total_ scores between the two groups and also between spastic and non-spastic patients, for the four studied speeds, when using 2 to 5 synergies as input to the NNMF algorithm.

In the case of the *r*^2^ coefficient, a minimum of 0.70 ± 0.05 (Figure 2BI) and 0.69 ± 0.06 (Figure 2BII) were obtained for the healthy and iSCI group, respectively, for the set of 8 muscles. Both values were obtained for the speed of 60 rpm. On the other hand, a maximum of 0.73 ± 0.09 and 0.72 ± 0.06 were obtained for the healthy and iSCI group, respectively. Both values were obtained for the speed of 30 rpm.

As it happened for VAF_total_, also *r*^2^ values decreased in the case of 13-muscle dataset. A minimum of 0.63 ± 0.08 (Figure 2BIII) and 0.61 ± 0.06 (Figure 2BIV) were obtained for the healthy (at 42 rpm) and iSCI group (at 60 rpm), respectively. A maximum of 0.67 ± 0.91 and 0.65 ± 0.07 were obtained for the healthy and iSCI group, respectively, both at 30 rpm.

The tests of equality of means from Independent Student's *t*-tests revealed no significant differences for the *r*^2^ scores between the two groups and also between spastic and non-spastic patients, for all speeds and input number of synergies.

#### Synergy vectors and activation coefficients

**Figure 3:**
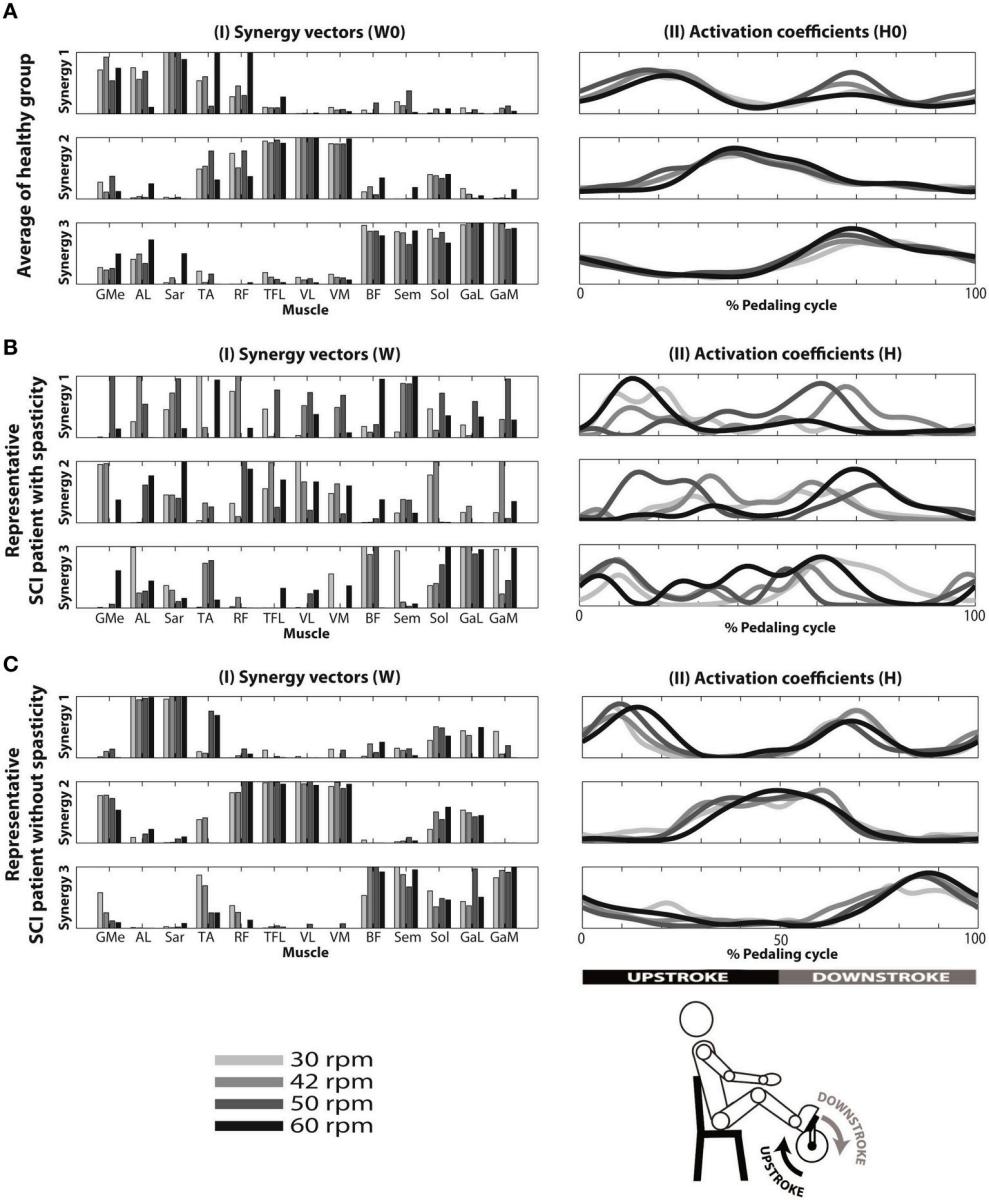
**Reconstruction of EMG envelopes in four speeds (30, 42, 50, and 60 rpm) using concatenated data from the 10 healthy subjects (A), and individual data from a patient with spasticity—ID 04 (B) and a patient without spasticity—ID 09 (C), applying the NNMF algorithm with three synergies**. I: muscle synergy vectors. Each muscle synergy vector has a time-invariant profile, representing the relative contribution of each synergy for each muscular pattern. Muscle synergy vectors were normalized by their maximum value. II: averaged activation coefficients, indicating time-variant profiles responsible to activate each synergy.

The reference sets of three muscle synergy vectors (W_0_) and the corresponding activation coefficients (H_0_) of the healthy group at four different speeds are represented in Figures 3AI,II, respectively. Synergy 1, activated predominantly during the upstroke phase of cycling (see Figure 3AII), was represented by the activity of GMed, AL, Sar, TA, and RF. Synergy 2, activated during the final upstroke phase and initial downstroke phase of cycling, was represented by the activity of TFL, VL, VM and, to a lower extent, TA and RF. Synergy 3, activated during the downstroke phase of cycling, was composed by the activity of BF, Sem, Sol, GaL, and GaM.

Results from a representative iSCI patient (ID 04) with spasticity are represented in Figures 3BI,II. High variability of muscle synergy vectors and activation coefficients can be observed across speeds. This is reflected in the values of normalized scalar product between muscle synergy vectors W extracted with the set of 13 muscles, and the reference matrices W_0_ (W · W_0_). This ranged from 0.41 to 0.69 in synergy 1, from 0.57 to 0.78 in synergy 2, and from 0.74 to 0.90 in synergy 3 (see Table 3). In the case of activation coefficients, H · H_0_ ranged from 0.63 to 0.91 for activation coefficient 1, from 0.59 to 0.90 for activation coefficient 2, and from 0.50 to 0.87 for activation coefficient 3. When only 8 muscles were considered, W · W_0_ ranged from 0.41 to 0.66 for synergy vector 1, from 0.70 to 0.73 for synergy vector 2, and from 0.74 to 0.96 for synergy vector 3 (see Table 4). Normalized scalar product ranged from 0.60 to 0.81 for activation coefficient 1, from 0.58 to 0.88 for activation coefficient 2, and from 0.61 to 0.90 for activation coefficient 3.

**Table 3:** **Normalized scalar product between matching muscle synergy vectors from matrices W of each patient and the matrix W_0_ obtained from all healthy subjects pooled together, considering the set of 13 muscles**.

**Patient ID**	**W** · **W**_**0**_ **at 30 rpm**			**W** · **W**_**0**_ **at 42 rpm**			**W** · **W**_**0**_ **at 50 rpm**			**W** · **W**_**0**_ **at 60 rpm**		
	**1**	**2**	**3**	**1**	**2**	**3**	**1**	**2**	**3**	**1**	**2**	**3**
1	0.48	0.71	0.79	0.64	0.67	0.78	0.60	0.78	0.68	0.74	0.68	0.78
2	**0.91**	0.89	**0.99**	**0.92**	0.81	**0.91**	0.87	0.73	**0.90**	0.87	0.79	0.88
3	0.55	0.83	**0.96**	0.58	0.84	**0.95**	0.40	0.78	**0.91**	0.46	0.73	**0.95**
4	0.62	0.78	**0.90**	0.61	0.68	0.74	0.69	0.57	0.85	0.41	0.71	0.79
5	0.79	0.88	**0.94**	0.78	0.80	**0.90**	0.75	0.77	**0.93**	**0.93**	**0.90**	0.88
6	0.73	0.54	0.73	0.45	0.58	0.67	0.73	0.65	0.61	0.67	0.76	0.76
7	**0.96**	**0.96**	**0.96**	**0.93**	**0.97**	**0.94**	0.88	0.77	0.68	0.86	0.89	0.85
8	0.70	0.69	0.86	0.50	0.75	0.85	0.70	0.31	0.66	0.74	0.39	0.78
9	0.76	**0.95**	0.83	0.68	**0.92**	0.83	0.81	0.89	**0.96**	0.57	0.88	0.85
10	0.54	0.80	**0.93**	0.71	0.78	**0.97**	0.44	0.83	0.85	0.40	0.88	0.82
Mean	0.71	0.80	0.89	0.68	0.78	0.85	0.69	0.71	0.80	0.67	0.76	0.83
(SD)	(0.13)	(0.10)	(0.07)	(0.12)	(0.09)	(0.08)	(0.12)	(0.12)	(0.12)	(0.16)	(0.11)	(0.05)

*The scalar product was abbreviated with the notation “W · W0.” Values ≥ 0.9 appear in bold*.

**Table 4:** **Normalized scalar product between matching muscle synergy vectors from matrices W of each patient and the matrix W_0_ obtained from all healthy subjects pooled together, considering the set of 8 muscles**.

**Patient ID**	**W** · **W**_**0**_ **at 30 rpm**			**W** · **W**_**0**_ **at 42 rpm**			**W** · **W**_**0**_ **at 50 rpm**			**W** · **W**_**0**_ **at 60 rpm**		
	**1**	**2**	**3**	**1**	**2**	**3**	**1**	**2**	**3**	**1**	**2**	**3**
1	0.37	0.74	0.84	0.73	0.67	0.71	0.69	0.87	0.81	0.59	0.78	0.75
2	0.83	0.73	**0.98**	0.87	0.72	0.86	0.82	0.51	0.87	**0.95**	0.70	0.84
3	0.80	0.76	**0.97**	0.79	0.81	**0.96**	0.81	0.89	**0.95**	0.68	0.83	**0.97**
4	0.66	0.73	**0.96**	0.60	0.70	0.86	0.46	0.72	0.82	0.41	0.71	0.74
5	0.76	0.73	**0.98**	0.73	0.65	0.78	0.39	0.74	0.82	**0.95**	0.83	0.70
6	0.36	0.58	0.71	0.52	0.89	0.85	0.79	0.86	0.69	0.56	0.83	0.88
7	0.68	0.87	0.81	**0.96**	**0.93**	**0.99**	0.70	0.56	0.87	**0.94**	0.75	**0.93**
8	0.54	0.70	0.78	0.84	**0.91**	**0.95**	**0.93**	0.57	0.81	**0.94**	0.79	0.85
9	0.75	0.89	0.88	0.70	0.81	**0.91**	0.77	0.85	**0.97**	0.75	0.76	**0.93**
10	0.22	0.80	0.83	0.12	0.88	0.85	0.15	0.88	0.78	0.23	0.78	0.84
Mean	0.60	0.75	0.87	0.69	0.80	0.87	0.65	0.74	0.84	0.70	0.78	0.84
(SD)	(0.18)	(0.06)	(0.08)	(0.16)	(0.09)	(0.06)	(0.19)	(0.12)	(0.06)	(0.20)	(0.04)	(0.07)

*The scalar product was abbreviated with the notation “W · W0.” Values ≥ 0.9 appear in bold*.

Results from a representative iSCI patient (ID 09) without spasticity are represented in Figures 3CI,II. In this case, muscle synergy vectors and activation coefficients were very similar across the speeds, as it happened in the healthy group. W · W_0_ ranged from 0.57 to 0.81 for synergy 1, from 0.88 to 0.95 for synergy 2, and from 0.83 to 0.96 for synergy 3 (see Table 3). In the case of activation coefficients, H · H_0_ ranged from 0.65 to 0.87 for activation coefficient 1, from 0.92 to 0.95 for activation coefficient 2, and from 0.78 to 0.92 for activation coefficient 3. For the set of 8 muscles, W · W_0_ ranged from 0.70 to 0.77 for synergy vector 1, from 0.76 to 0.89 for synergy vector 2, and from 0.88 to 0.97 for synergy vector 3 (see Table 4). As for the activation coefficients, H · H_0_ ranged from 0.78 to 0.95 for synergy 1; 0.91 to 0.97 for synergy 2; 0.72 to 0.93 for synergy 3.

When comparing these similarity values between spastic and non-spastic patients, most of the metrics presented a lower mean in spastic patients, despite those differences were not statistically significant. Just in the case of H2 · H_0_2 at 30 rpm for the dataset of 13 muscles, differences were significant (*p* = 0.036), with spastic patients (0.9 ± 0.04) presenting lower similarity than non-spastic (0.94 ± 0.02).

Normalized scalar products between synergy vector 3 (W3) from each iSCI patients and the corresponding synergy vector from the healthy group (W_0_3) were, on average, higher than those obtained for W1 and W2, for all speeds (see Tables 3, 4), in both sets of 13 and 8 muscles. In general, normalized scalar products were higher at lower speeds. Also, normalized scalar products using the set of 13 muscles were higher than those observed with the set of 8 muscles at 30 rpm, and lower for the other speeds.

Normalized scalar products of activation coefficient 3 from each iSCI patient (H3) and activation coefficient 3 from the healthy group (H_0_3) were, on average, higher than those obtained for H1 and H2, for all speeds and sets of muscles.

### Correlation with clinical metrics

#### Correlation with gait performance scores

**Figure 4:**
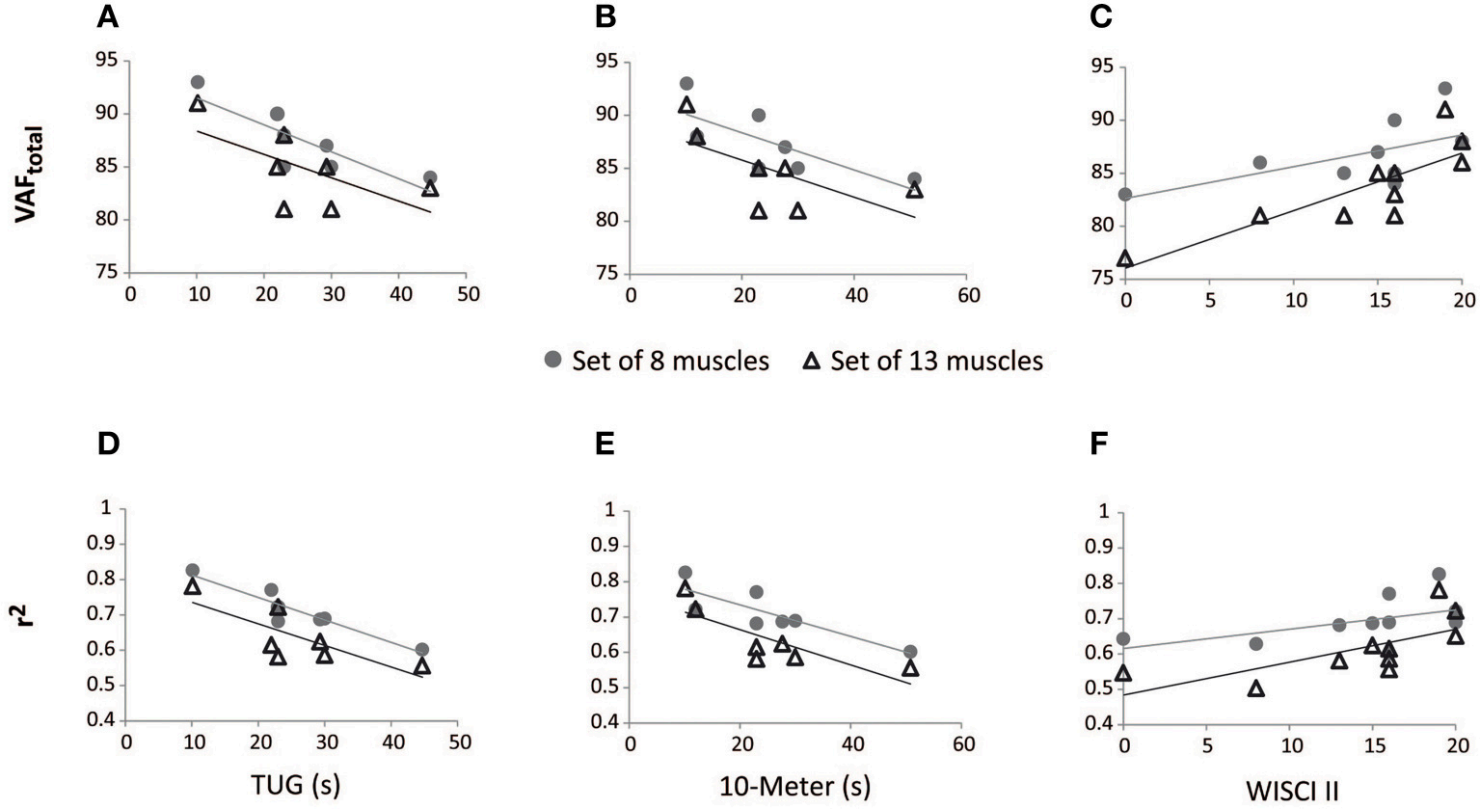
**Correlation between walking tests scores in iSCI patients and reconstruction goodness indexes at 42 rpm, for the sets of 8 and 13 muscles**. VAF_total_ scores correlated negatively with TUG **(A)** and 10-m **(B)** tests; VAF_total_ scores correlated positively with WISCI II **(C)**. *r*^2^ scores correlated negatively with TUG **(D)** and 10-m **(E)** tests; *r*^2^ scores correlated positively with WISCI II **(F)**.

Spearman's rank-order correlations were run to determine the relationship between reconstruction goodness scores (VAF_total_ and *r*^2^) and clinical measurements of gait performance of iSCI patients. There was a strong, positive correlation between EMG reconstruction goodness scores when using the set of 13 muscles and WISCI II scores, which was statistically significant (*r*_s_ > 0.75, *p* < 0.05; see Table 5). Significant correlations were also found between WISCI II and VAF_total_ and *r*^2^ for the speed 42 rpm, when using the set of 8 muscles. Two examples of the correlation between WISCI II and reconstruction goodness indexes (VAF_total_ and *r*^2^) at 42 rpm are represented in Figures 4C,F, respectively.

**Table 5:** **Correlation of gait scales (from iSCI patients) with VAF_total_ and *r*^2^ results of the EMG reconstruction with 8 and 13 muscles, for all speeds**.

		**8 Muscles**				**13 Muscles**			
		**30 rpm**	**42 rpm**	**50 rpm**	**60 rpm**	**30 rpm**	**42 rpm**	**50 rpm**	**60 rpm**
VAF_total_	TUG	*r* = 0.029	***r*** = −0.833^*^	*r* = 0.124	*r* = 0.076	*r* = −0.115	*r* = −0.629	*r* = 0.174	*r* = −0.121
		*p* = 0.950	***p* = 0.020**	*p* = 0.791	*p* = 0.871	*p* = 0.806	*p* = 0.130	*p* = 0.709	*p* = 0.796
	10-m	*r* = −0.001	*r* = −0.739	*r* = 0.167	*r* = 0.079	*r* = −0.219	*r* = −0.643	*r* = 0.078	*r* = −0.243
		*p* = 0.998	*p* = 0.058	*p* = 0.720	*p* = 0.866	*p* = 0.637	*p* = 0.119	*p* = 0.868	*p* = 0.599
	WISCI II	*r* = 0.472	*r* = 0.650^*^	*r* = 0.350	*r* = 0.402	*r* = 0.845^**^	*r* = 0.850^**^	*r* = 0.809^**^	*r* = 0.749^*^
		*p* = 0.168	***p* = 0.042**	*p* = 0.322	*p* = 0.250	***p* = 0.002**	***p* = 0.002**	***p* = 0.005**	***p* = 0.013**
*r*^2^	TUG	*r* = −0.002	***r*** = −0.934^**^	*r* = −0.094	*r* = −0.082	*r* = −0.235	***r*** = −0.777^*^	*r* = −0.115	*r* = −0.420
		*p* = 0.997	***p* = 0.002**	*p* = 0.841	*p* = 0.862	*p* = 0.612	***p* = 0.040**	*p* = 0.806	*p* = 0.348
	10-m	*r* = −0.057	***r*** = −0.843^*^	*r* = −0.039	*r* = −0.055	*r* = −0.383	***r*** = −0.817^*^	*r* = −0.204	*r* = −0.541
		*p* = 0.904	***p* = 0.017**	*p* = 0.934	*p* = 0.907	*p* = 0.397	***p* = 0.025**	*p* = 0.661	*p* = 0.210
	WISCI II	*r* = 0.542	*r* = 0.677^*^	*r* = 0.412	*r* = 0.228	*r* = 0.899^**^	*r* = 0.825^**^	*r* = 0.825^**^	*r* = 0.794^**^
		*p* = 0.106	***p* = 0.032**	*p* = 0.236	*p* = 0.527	***p* = 0.000**	***p* = 0.003**	***p* = 0.003**	***p* = 0.006**

*Significant correlation values (p < 0.05) appear in bold*.

* *Correlation is significant at the 0.05 level (2-tailed)*.

** *Correlation is significant at the 0.01 level (2-tailed)*.

The *r*^2^ index correlated negatively with TUG and 10-m tests, in both cases of 13 and 8 muscles, for 42 rpm, as represented in Table 5 and Figures 4D,E. VAF_total_ with 8 muscles also presented significant correlation with TUG scores, at 42 rpm. The correlation of 10-m test with VAF_total_ presented a *p*-value of 0.058, for the set of 8 muscles. Correlations between VAF_total_ and TUG and 10-m tests, at 42 rpm, are presented in Figures 4A,B, respectively.

#### Correlation with spasticity measures

**Figure 5:**
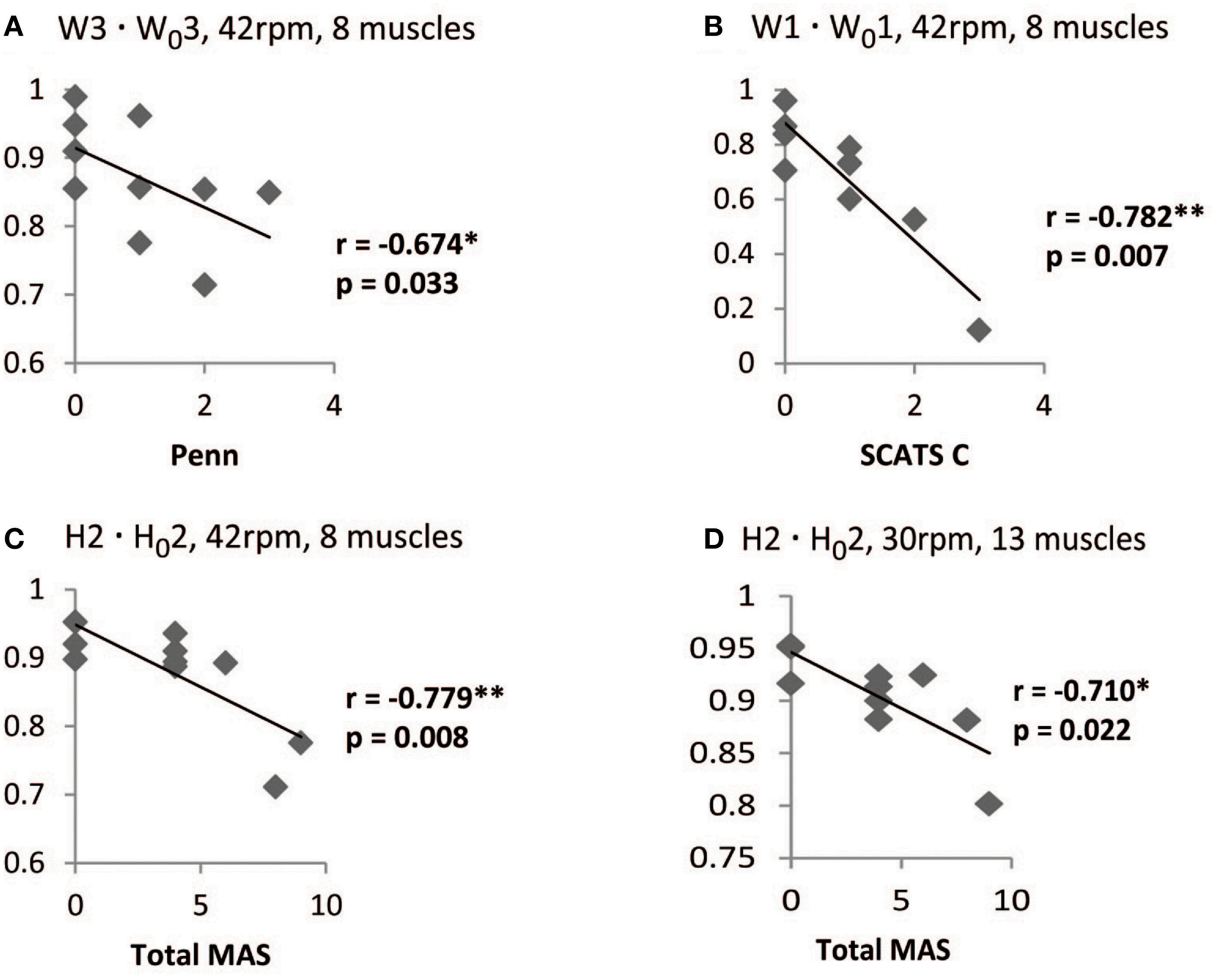
**Correlation between spasticity scales in iSCI patients and W · W_0_, and H · H_0_ scores**. For the set of 8 muscles at 42 rpm, W3 · W_0_3 correlated negatively with PENN **(A)** and W1 · W_0_1 correlated negatively with clonus spasms assessed with SCATS **(B)**. H2 · H_0_2 correlated negatively with Ashworth for the sets of 8 muscles at 42 rpm **(C)** and 13 muscles at 30 rpm **(D)**. ^*^Correlation is significant at the 0.05 level (2-tailed). ^**^Correlation is significant at the 0.01 level (2-tailed).

Significant correlations were found, especially for the set of 8 muscles, with the Penn scale. For instance, W1 · W_0_1 correlated negatively (*p* < 0.05, *r*_s_ < 0) with Penn scale for all speeds, except for 50 rpm (*p* = 0.076; see Table 6). W3 · W_0_3 correlated negatively (*p* < 0.05, *r*_s_ < 0) with Penn scale for 42 and 50 rpm speeds. The trend found when correlating Penn with W3 · W_0_3 at 42 rpm is represented in Figure 5A. In this case, patients with higher Penn scores presented lower similarity on W3 · W_0_3.

**Table 6:** **Correlation of spasticity scales and W · W_0_, for both sets of 8 and 13 muscles, for all speeds**.

		**30 rpm**			**42 rpm**			**50 rpm**			**60 rpm**		
		**W1**	**W2**	**W3**	**W1**	**W2**	**W3**	**W1**	**W2**	**W3**	**W1**	**W2**	**W3**
8 muscles	Total MAS	*r* = −0.342	*r* = −0.424	*r* = −0.146	*r* = −0.494	*r* = −0.222	*r* = −0.329	*r* = −0.513	*r* = 0.291	*r* = −0.462	*r* = −0.519	*r* = 0.329	*r* = −0.367
		*p* = 0.333	*p* = 0.221	*p* = 0.688	*p* = 0.147	*p* = 0.538	*p* = 0.353	*p* = 0.129	*p* = 0.414	*p* = 0.178	*p* = 0.124	*p* = 0.353	*p* = 0.296
	Penn	***r*** = −0.642^*^	*r* = −0.178	*r* = −0.191	***r*** = −0.756^*^	*r* = −0.267	***r*** = −0.674^*^	*r* = −0.585	***r*** = − 0.750^*^	***r*** = −0.636^*^	***r*** = −0.756^*^	*r* = 0.4	*r* = −0.375
		***p* = 0.045**	*p* = 0.623	*p* = 0.598	***p* = 0.011**	*p* = 0.456	***p* = 0.033**	*p* = 0.076	***p* = 0.012**	***p* = 0.048**	***p* = 0.011**	*p* = 0.251	*p* = 0.286
	SCATS C	*r* = −0.575	*r* = −0.239	*r* = −0.162	***r*** = −0.782^**^	*r* = −0.213	*r* = −0.575	*r* = −0.569	***r*** = −0.724^*^	*r* = −0.614	***r*** = −0.750^*^	*r* = 0.465	*r* = −0.33
		*p* = 0.082	*p* = 0.506	*p* = 0.656	***p* = 0.007**	*p* = 0.554	*p* = 0.082	*p* = 0.086	***p* = 0.018**	*p* = 0.059	***p* = 0.012**	*p* = 0.175	*p* = 0.352
	SCATS F	*r* = −0.087	*r* = −0.609	*r* = −0.087	*r* = −0.261	*r* = −0.174	*r* = −0.435	*r* = −0.174	*r* = 0.087	*r* = −0.348	*r* = 0.087	*r* = 0.609	*r* = −0.261
		*p* = 0.811	*p* = 0.062	*p* = 0.811	*p* = 0.466	*p* = 0.631	*p* = 0.209	*p* = 0.631	*p* = 0.811	*p* = 0.324	*p* = 0.811	*p* = 0.062	*p* = 0.466
	SCATS E	*r* = −0.437	*r* = 0.007	*r* = 0.146	*r* = −0.562	*r* = −0.52	***r*** = −0.694^*^	***r*** = −0.874^**^	*r* = 0.347	*r* = −0.409	*r* = −0.499	*r* = 0.076	***r*** = −0.791^**^
		*p* = 0.207	*p* = 0.985	*p* = 0.688	*p* = 0.091	*p* = 0.123	***p* = 0.026**	***p* = 0.001**	*p* = 0.326	*p* = 0.24	*p* = 0.142	*p* = 0.834	***p* = 0.006**
13 muscles	Total MAS	*r* = −0.158	*r* = −0.386	*r* = −0.253	*r* = −0.266	*r* = −0.507	*r* = −0.374	*r* = −0.234	*r* = −0.215	*r* = −0.279	*r* = −0.196	*r* = 0.070	*r* = −0.310
		*p* = 0.662	*p* = 0.270	*p* = 0.480	*p* = 0.457	*p* = 0.135	*p* = 0.287	*p* = 0.514	*p* = 0.550	*p* = 0.435	*p* = 0.587	*p* = 0.848	*p* = 0.383
	Penn	***r*** = −0.706^*^	*r* = −0.559	*r* = −0.407	*r* = −0.305	***r*** = −0.636^*^	*r* = −0.083	***r*** = −0.686^*^	*r* = 0.267	*r* = −0.165	*r* = −0.496	*r* = −0.14	*r* = −0.337
		***p* = 0.023**	*p* = 0.093	*p* = 0.243	*p* = 0.391	***p* = 0.048**	*p* = 0.82	***p* = 0.028**	*p* = 0.456	*p* = 0.648	*p* = 0.145	*p* = 0.7	*p* = 0.341
	SCATS C	*r* = −0.608	*r* = −0.53	*r* = −0.33	*r* = −0.336	*r* = −0.582	*r* = −0.045	***r*** = −0.659^*^	*r* = 0.181	*r* = −0.123	*r* = −0.53	*r* = −0.052	*r* = −0.272
		*p* = 0.062	*p* = 0.115	*p* = 0.352	*p* = 0.342	*p* = 0.078	*p* = 0.901	***p* = 0.038**	*p* = 0.617	*p* = 0.735	*p* = 0.115	*p* = 0.887	*p* = 0.448
	SCATS F	*r* = 0.261	*r* = −0.261	*r* = −0.261	*r* = −0.174	*r* = −0.348	*r* = −0.348	*r* = 0.174	*r* = −0.174	*r* = −0.087	*r* = 0.348	*r* = 0.348	*r* = −0.174
		*p* = 0.466	*p* = 0.466	*p* = 0.466	*p* = 0.631	*p* = 0.324	*p* = 0.324	*p* = 0.631	*p* = 0.631	*p* = 0.811	*p* = 0.324	*p* = 0.324	*p* = 0.631
	SCATS E	*r* = −0.534	*r* = −0.201	*r* = −0.111	*r* = 0.166	*r* = −0.388	*r* = 0.062	*r* = −0.472	*r* = 0.284	*r* = 0.139	*r* = −0.312	*r* = 0.042	*r* = −0.139
		*p* = 0.112	*p* = 0.577	*p* = 0.76	*p* = 0.646	*p* = 0.267	*p* = 0.864	*p* = 0.169	*p* = 0.426	*p* = 0.702	*p* = 0.38	*p* = 0.909	*p* = 0.702

*Significant correlation values (p < 0.05) appear in bold*.

* *Correlation is significant at the 0.05 level (2-tailed)*.

** *Correlation is significant at the 0.01 level (2-tailed)*.

*W1, muscle synergy vector 1; W2, muscle synergy vector 2; W3, muscle synergy vector 3*.

Some correlations with clonus and extensor spasms assessed with SCATS were also significant (*p* < 0.05) or presented *p*-values between 0.05 and 0.1 for some of the similarity scores and speeds, for the set of 8 muscles. The trend found when correlating clonus from SCATS with W1 · W_0_1 at 42 rpm is represented in Figure 5B. In this case, patients with higher SCATS scores presented lower similarity on W1 · W_0_1.

Total MAS correlated negatively (*p* < 0.05, *r*_s_ < −0.75) with H2 · H_0_2 at 30 rpm, for the set of 8 muscles. The trend found when correlating Total MAS with H2 · H_0_2 at 42 rpm and 30 rpm for the sets of 8 and 13 muscles, respectively, is represented in Figures 5C,D. For both cases, patients with higher Total MAS scores presented lower similarity on H2 · H_0_2.

## Discussion

The results of this work support the hypothesis that iSCI patients preserve a synergistic control of cycling, being this evidence not reported previously in the literature. The second relevant finding of this work is that gait motor performance and spasticity of iSCI patients correlate with muscle synergies outcomes extracted during cycling movements. In particular, synergy reconstruction goodness scores (VAF, *r*^2^) correlate with walking performance scales scores, whereas the degree of similarity between iSCI and healthy synergies appears to be correlated with spasticity scales scores, especially the spasms frequency measured by Penn scale. The next section discusses these findings in more detail, providing additional reasoning for their correct interpretation.

### Muscle synergy analysis

#### Electromyographic patterns in iSCI patients during cycling

With the exception of TA, VM, GaL, and Sol, the average EMG envelopes of all muscles presented very similar activation timing and shape between the two groups, along all matched speeds. In the case of the three aforementioned distal muscles (TA, GaL, and Sol), which are usually affected by spasticity (Bravo-Esteban et al., 2013) and co-activation (Gómez-Soriano et al., 2010), we hypothesize that the differences in the average activation timing and shape could be explained by the hypertonia and clonus presented by some of the patients recruited.

Several studies have previously showed that the EMG activity of lower limb muscles is similar between sides, during tasks like walking and cycling in healthy subjects (Clark et al., 2010; Gizzi et al., 2011; Hug et al., 2011). For this reason, we analyzed only the dominant leg of healthy participants. In the case of iSCI patients, we considered the most affected side, in order to extract more relevant information on the underlying impairments.

In addition to the eight muscles studied previously by us in healthy subjects (Barroso et al., 2014), five additional muscles (mono and bi-articular) have been recorded to increase the sample number and to test the influence of the number of muscles required to correlate synergy metrics with clinical scales.

As part of the standard rehabilitation program of the hospital, patients were already trained in the use of MOTOmed viva2. Therefore, we did not expect any temporal effect on muscle activation.

#### Reconstruction goodness scores

To assess the reconstruction goodness of EMG patterns with a given number of synergies, we used two different coefficients: VAF_total_ and coefficient of determination (*r*^2^). In our previous study (Barroso et al., 2014), we obtained VAF_total_ values of approximately 90% using three synergies, to reconstruct a set of eight lower limb muscles in eight healthy subjects, for cycling speeds ranging from 43 ± 2.7 to 70 ± 4.0 rpm. In this study, similar VAF_total_ values (86.7 ± 2.0 to 88.5 ± 4.2%) were obtained for the same set of muscles in ten healthy subjects. As expected, both VAF_total_ and *r*^2^ values decreased when using three synergies to reconstruct the set of 13 muscles, when compared with the case of 8 muscles. As referred by Steele et al. (2013), Clark et al. (2010), and Monaco et al. (2010), the higher the number of muscles, the lower is the reconstruction goodness score. No significant differences were found in the VAF_total_ and *r*^2^ scores neither between the iSCI group and the healthy group nor between spastic and non-spastic patients, for the 4 speeds, when using 2 to 5 synergies. This result may be explained by the low complexity of cycling (few degrees of freedom), which seems to be executed by the same number of synergies by both healthy and iSCI subjects.

Differently from previous studies, in which VAF is used to define the optimal number of synergy for each subject, we introduced a global criterion (VAF_total_ values ≥ 85% in at least 50% of the subjects) in order to fix a “globally optimum” number of synergies for all subjects. This criterion allowed us to (i) use VAF_total_ values as a continuous quantitative metric of motor performance, and (ii) perform a direct comparison between patients' synergies and a standardized reference dataset (W_0_ and H_0_).

#### Similarity of synergy vectors and activation coefficients

Little is known about the effect of iSCI on muscle synergies (Ivanenko et al., 2009). The few studies that investigated this synergistic control focused on gait (Ivanenko et al., 2003, 2009; Hayes et al., 2014). These studies suggested that the training post-SCI and the underlying plasticity lead to a reorganization of interneuronal networks, this way modifying and creating new muscle synergies. Notwithstanding, the synergistic control of iSCI patients during cycling has not been described yet, to our best knowledge.

Interestingly, the activation coefficients when using the set of 13 muscles (see Figure 3AII) were very similar to those obtained with the set of 8 muscles, and similar to those already published by our group (Barroso et al., 2014). Synergy vectors were also very similar between the 8- and 13-muscle datasets, with the additional five muscles being incorporated within the three synergy vectors (see Figure 3AI). Synergy 1 (involving primarily hip flexor, knee extensor, and ankle dorsiflexor) mainly provided force to start the upstroke phase of cycling in healthy subjects. Synergy 2 (hip abductor, hip flexor, knee extensors and ankle plantarflexor) contributed to the second part of upstroke phase and also to the initial downstroke phase. Synergy 3 (hip extensors, knee flexors and ankle plantarflexors) activated muscles responsible for downstroke phase of cycling.

In the healthy participants, antagonist muscles were not activated by the same synergy (e.g., the quadriceps and hamstrings, the TA and the Triceps Surae), in accordance with the literature on walking (Clark et al., 2010) and cycling (Hug et al., 2011). As expected, patients with no diagnosed spasticity presented similar synergy vectors to the healthy people (levels of co-activation of agonists/antagonists very low; see representative patient from Figure 3CI). However, this was not the case of some iSCI patients diagnosed with spasticity (see representative patient from Figure 3BI). In this case, there was high variability of muscle synergy vectors and activation coefficients across the speeds, and also antagonist muscles were activated by the same synergy (e.g., TA and Sol activated by synergy 3 in Figure 3BI). Although spastic patients presented, on average, lower similarity values (W · W0 and H · H0) than non-spastic patients, differences were not statistically significant, except for the case of H2 · H_0_2 at 30 rpm for the dataset of 13 muscles. This metric could also predict Total MAS scores.

In general, muscle synergies composition of more impaired patients was less similar to the healthy controls. We speculate that patients with poor motor condition may have created maladaptive plasticity that modified the existing muscle synergies. When considering the whole iSCI group, similarity with the healthy reference set was higher for the activation coefficients than for the synergy vectors. We hypothesized that these results may indicate less disruption of the corticospinal drive (represented by activation coefficients) than the disruption of the spinal organization (represented by the synergy vectors) after SCI (Ivanenko et al., 2003; Ting et al., 2015).

Synergy 3 (activated during the downstroke phase of cycling) from iSCI patients best correlated with the healthy group, for both spastic and non-spastic patients. This has been observed in the synergy vectors (W3 · W_0_3) and activation coefficients (H3 · H_0_3), indicating less variability for this synergy composition and activation. Therefore, we hypothesized that the similarity scores for W1 and W2, as well as H1 and H2, which present lower correlation values with the healthy group, would better distinguish the spasticity levels of each patient.

### Correlation with clinical metrics

#### Predictions of gait performance

Gait speed is an important outcome variable in clinical assessment. The criteria used to include similar patients in a group are usually based on classic clinical evaluations and walking speed (Nadeau et al., 2011). Clark et al. (2010) reported that the number of muscle synergies in post-stroke patients correlated with the preferred walking speed. Also, Routson et al. (2013) referred that those post-stroke patients that improved the activation coefficients (more similar to the healthy group) also improved walking performance. van Hedel et al. (2005) reported that the 10-m test was more sensitive than the WISCI II in demonstrating improvements in walking performance in iSCI subjects. Based on these observations, and also on our previous findings on similarity of muscle synergies between cycling and walking (Barroso et al., 2014), we hypothesized that the analysis of muscle synergies during cycling correlates with performance scales related to gait speed, such as 10-m and TUG tests.

Our results showed positive significant correlations between WISCI II and EMG reconstruction goodness scores (VAF_total_ and *r*^2^), when using the set of 13 muscles (see Table 5). In the case of gait speed tests, *r*^2^ index correlated negatively with TUG and 10-m tests, in both cases of 13 and 8 muscles. These results are in accordance with the fact that patients with good walking performance (lower time to perform TUG and 10-m test) and lower amount of assistance needed (higher scores in the WISCI II scale) present higher signal-to-noise ratio than those with poor walking performance, as severely impaired subjects usually present reduced signal-to-noise ratio in the EMG signals due to reduced signal strength (Lee et al., 2011). As a consequence of lower signal-to-noise, lower VAF_total_ and *r*^2^ values are expected (Steele et al., 2013).

In addition, 42 rpm seems to be the most appropriate speed to assess motor performance in iSCI patients.

#### Predictions of spasticity

The difficulty to classify a subject as spastic or not is a well-known problem (Reichenfelser et al., 2012). Despite the valuable information of pathophysiological mechanisms involved in spasticity (Biering-Sørensen et al., 2006), there is still need for novel tools capable of providing quantitative metrics of spasticity, with low intra and inter-rater variability (Gómez-Soriano et al., 2012). In post-stroke patients, spasticity is characterized by high levels of muscle tone and a relative absence of spasms, whereas in iSCI patients, spasticity is mainly associated with the presence of flexor and extensor spasms triggered by cutaneous stimulation (Bennett, 2008). Ashworth and modified Ashworth scales are commonly used to assess spasticity, although they specifically measure hypertonia (Gómez-Soriano et al., 2012). It has been previously suggested to combine them with a spasms frequency scale to obtain more information of the spasticity of the iSCI patient (Priebe et al., 1996).

Significant correlations were obtained when correlating similarity of synergy vectors (W · W_0_) with the spasms frequency Penn scale, especially for the dataset of 8 muscles. W1 · W_0_1 was the one with better correlation values with Penn scale. Also, some correlations with clonus and extensor spasms assessed with SCATS were also significant (*p* < 0.05) or presented *p*-values between 0.05 and 0.1 for some of the synergy vectors and speeds.

Significant correlations were found between Total MAS scores and H2 · H_0_2 at low speeds. This indicates that H2 · H_0_2 at low speeds may be useful to assess the level of hypertonia, whereas similarity of synergy vectors may be used to assess the spasms frequency. On the other hand, these results also encourage a wider use of Penn and SCATS scale to assess spasticity syndrome in iSCI patients.

## Conclusion

The primary goal of this study was to describe the synergistic motor control of iSCI patients during cycling. Results showed that SCI patients preserve a synergistic control of lower limb muscles during cycling movements, evidence that was not reported previously in the literature.

As a secondary goal, we positively tested the ability of muscle synergy analysis during cycling movements to assess walking functionality, as well as to quantify hypertonia and spasms, which are important clinical conditions of spasticity present in iSCI patients (Bennett, 2008).

Additionally, we explored the sensitivity of the method to changes in speeds and number of muscles, being this information relevant for efficient applicability in everyday clinical practice. We found out that the analysis of 8 muscles was sufficient to quantitatively assess spasticity of the lower limbs and also gait performance, and that 42 rpm appears to be the most convenient cycling speed for such analysis.

As future work, we are planning to test intra and inter-rater variability of this tool, as well as to follow-up iSCI patients during at least 9 months after the injury, in order to validate this approach for long term monitoring and assessment of different therapeutic interventions. Further application of this tool by clinicians is also needed to facilitate consensus amongst researchers on the use of the analysis of muscle activity (and coordination) to extract quantitative metrics of neurologically induced motor impairments.

## Funding

This study was funded by the Spanish Ministry for Science and Innovation, in the framework of the project HYPER (CONSOLIDER-INGENIO 2010) “Hybrid Neuroprosthetic and Neurorobotic Devices for Functional Compensation and Rehabilitation of Motor Disorders” (Ref. CSD2009-00067) and the Spanish project ASSOCIATE (Ref. DPI2014-58431-C4-1-R).

### Conflict of interest statement

The authors declare that the research was conducted in the absence of any commercial or financial relationships that could be construed as a potential conflict of interest.
